# The Ether Douche or Lavement for Local Pain

**Published:** 1884-01-20

**Authors:** C. H. Hughes


### THE ETHER DOUCHE OR LAVEMENT FOR LOCAL PAIN.

By C. H. Hughes, M.D.

A paragraph in the Medical Times of the 10th of February, 1883, referring to the ether spray in the cure of neuralgia, prompts me to call attention to the fact that ether lavements have been employed by me in all painful surface affections for many years, whether with or without inflammation, but mainly in neuralgic affections. In facial, sciatic and cervical neuralgias, no remedy except galvanism has given me such signal satisfaction during the past ten years of my practice iu neurology. These lavements will cure some cases of recent origin; they will relieve all. I use the ether douche, not the spray, and Dr. McLane Hamilton is in error in his reference to my treatment of the intense pain of cerebellar abscess by ether spray. In the case referred to, which I reported in 1877 (Journal of Mental and Nervous Diseases, October), I simply poured the ether on the head so copiously as to benumb all sensibility and restore a state of ease and mental tranquillity to a patient absolutely maddened with pain.

The ether-douche or lavement in trigeminal neuralgia is quite uncomfortable to many persons, on account of the unpleasant impression of the ether on the nose and eyes; and when applied to the supra-orbital region great care should be taken to keep the ether out of the eyes by laying the head back and covering the eyes with a handkerchief. If the ether should get in the eyes, the patient should be cautioned not to rub them, but simply to sponge the eyes with cold water and wait patiently till the ether evaporates. The same is true in regard to ether getting in the ears.

There is no need of a spray apparatus for ether. It should be used freely in quantities adequate to the effect desired. It should be poured on the part till relief is obtained. I apply it in this way to the motor regions of the head and down the spine in general or unilateral chorea likewise.

No better agent can be employed for cephalalgia and for acute muscular tremor than the ether douche or lavement. Of late years I have heard of the ether spray, but the ether *douche* or lavement has been with me a most common and efficient agent in the local therapy of pain, especially superficial pain, for more than a decade, ranking with electricity, and better than mechanical vibration.—*Med. Times.*

